# A metabolic model of the mitochondrion and its use in modelling diseases of the tricarboxylic acid cycle

**DOI:** 10.1186/1752-0509-5-102

**Published:** 2011-06-29

**Authors:** Anthony C Smith, Alan J Robinson

**Affiliations:** 1 The Medical Research Council, Mitochondrial Biology Unit, Hills Road, Cambridge CB2 0XY, UK

## Abstract

**Background:**

Mitochondria are a vital component of eukaryotic cells and their dysfunction is implicated in a large number of metabolic, degenerative and age-related human diseases. The mechanism or these disorders can be difficult to elucidate due to the inherent complexity of mitochondrial metabolism. To understand how mitochondrial metabolic dysfunction contributes to these diseases, a metabolic model of a human heart mitochondrion was created.

**Results:**

A new model of mitochondrial metabolism was built on the principle of metabolite availability using MitoMiner, a mitochondrial proteomics database, to evaluate the subcellular localisation of reactions that have evidence for mitochondrial localisation. Extensive curation and manual refinement was used to create a model called *i*AS253, containing 253 reactions, 245 metabolites and 89 transport steps across the inner mitochondrial membrane. To demonstrate the predictive abilities of the model, flux balance analysis was used to calculate metabolite fluxes under normal conditions and to simulate three metabolic disorders that affect the TCA cycle: fumarase deficiency, succinate dehydrogenase deficiency and α-ketoglutarate dehydrogenase deficiency.

**Conclusion:**

The results of simulations using the new model corresponded closely with phenotypic data under normal conditions and provided insight into the complicated and unintuitive phenotypes of the three disorders, including the effect of interventions that may be of therapeutic benefit, such as low glucose diets or amino acid supplements. The model offers the ability to investigate other mitochondrial disorders and can provide the framework for the integration of experimental data in future studies.

## Background

Due to the involvement of the mitochondrion in many essential metabolic processes it is implicated in a wide and growing variety of disorders and pathologies [1]. However, it is often difficult to elucidate how a disease phenotype relates to the underlying genetic cause, as these disorders have complicated and non-intuitive phenotypes due to the inherent complexity of mitochondrial metabolism. A change in a small part of a complex network can affect the overall capabilities of a system, such as metabolite production, which can be impossible to predict without a systemic model. Models of metabolism allow these disorders to be studied by simulating the effects of a change in enzyme metabolism at a systems level and modelling these complex phenotypes as the consequence of changes in metabolic fluxes. Several models have been created that have attempted to represent the mitochondrion in its entirety [2-5], although these reconstructions are far from complete. Their construction has been hindered by the lack of a defined mitochondrial proteome, as available localisation data is difficult to query and use in conjunction with metabolic pathway data. Therefore these models have been limited to well-established pathways of the mitochondrion. These models are unlikely to be representative of the full metabolic capabilities of the mitochondrion, which may result in difficulties in modelling metabolic disorders that often have complicated and unintuitive phenotypes caused by complex interactions between reactions that may not otherwise be obviously interlinked. Evaluating the mitochondrial localization of thousands of reactions and providing a large list of additional reactions and metabolites is enabled by MitoMiner [6], a mitochondrial proteomic database that integrates experimental proteomic localization data with annotation from public resources and metabolic pathway data. The data and functionality of MitoMiner allows the creation of a more comprehensive metabolic model and provides a new opportunity to investigate mitochondrial metabolism.

Here we describe the construction of a model of mitochondrial metabolism in human heart cells called *i*AS253 by using the MitoMiner database and other public resources. We demonstrate that by using flux balance analysis (FBA) normal mitochondrial metabolism can be successfully simulated. The FBA method has been described in detail elsewhere [7,8], but briefly the main data required is a stoichiometric matrix (S), which represents all the reactions and metabolites in the model. The matrix is used with the assumption that the model is at a quasi steady state, represented as S • ν = 0, where ν is a flux distribution vector. Capacity and directionality constraints are then included by defining upper and lower bounds (ν_min _≤ ν ≤ ν_max_) of individual reactions. Directionality is incorporated by setting either the upper or lower bound of a reaction to zero. Although these constraints limit the number of possible metabolic flux distributions that satisfy the steady state, a very large number of possible solutions still exist, so a unique solution representing a flux distribution for the model is found by using an objective function. The objective function is a set of criteria chosen to match the biological objective of the system *in vivo *and an optimal flux distribution is found that maximises (or minimises) this function by using linear programming. If the objective function of the model matches the biological objective of the system, the optimal flux distribution should correspond with the flux distribution *in vivo*. For example a common objective function used for mitochondrion metabolism is maximum ATP production, so to reflect the primary role of the mitochondrion in cellular energy production.

The benefits of the FBA approach are that detailed kinetic information such as concentrations are not required, allowing the simulation of large models that would be infeasible using alternative methods. This makes the technique particularly attractive in biomedicine, where a phenotype can be the result of many interacting reactions and detailed kinetic information for each enzyme involved under disease conditions is unavailable and difficult to obtain experimentally. Furthermore, the models can be used to discover and assess the effect of interventions on a disease state that may restore the system to a normal state and thus be of therapeutic value.

In our new model we account for the most recent stoichiometric information of the ATP synthase [9], thermodynamic constraints and reaction directionality, and the change in protonation state between the mitochondrial matrix and the cytosol due to the difference in pH between these compartments. These are factors that have not all been accounted for in previous flux balance analysis simulations of mitochondrial metabolism. Having developed a model that simulates normal mitochondrial metabolism, we demonstrate the predictive capabilities of our new model, by investigating three TCA cycle disorders that affect core mitochondrial metabolism.

The first disorder, fumarase deficiency (OMIM: 606812), is caused by impairment of the fumarate hydratase enzyme, which converts fumarate into malate in the TCA cycle. The condition is exceptionally rare with around 40 cases reported globally to date [10]. The effects of the disorder are acute and include developmental delay, severe mental retardation, language impairment, seizures and dysmorphic facial features [10-12]. No medical treatments currently exist and most patients do not survive past early childhood [12]. In most of these cases there is almost no residual enzyme activity [13,14]. The diagnostic markers for this disorder are the presence of fumarate and 2-oxoglutarate in the urine, and lactate in the cerebrospinal fluid [13].

The second disorder, succinate dehydrogenase deficiency (OMIM: 252011) affects mitochondrial complex II, which links the TCA cycle with the electron transport chain by coupling the conversion of succinate to fumarate to the quinone pool. In cases of isolated succinate dehydrogenase deficiency the phenotype is highly variable and can include Leigh syndrome [15-17], leukodystrophy [16,17], Kearns-Sayre syndrome [18], cardiomyopathy [19] and mental and motor skill deterioration [20]. The onset of symptoms can be from childhood to adulthood and appears to correlate with residual enzyme activity, with those with higher complex II activity having milder symptoms and diagnosed later in life [21]. The metabolic profile of the disorder is elevated levels of lactate in blood plasma [16-18,20], and in some cases oxoglutarate and succinate is present in the urine [22] and succinate accumulates in brain [15].

The third disorder, α-ketoglutarate dehydrogenase deficiency (OMIM: 203740) affects the conversion of oxoglutarate to succinyl-CoA in the TCA cycle. The condition is extremely rare with only 23 cases recorded in the literature [23]. Onset of the disorder occurs shortly after birth and is characterised by encephalopathy and hyperlactatemia resulting in death in early childhood. The metabolic profile of the disorder is the excretion of oxoglutarate [24,25] and in many cases lactate [25,26]. The α-ketoglutarate dehydrogenase complex consists of three protein subunits; E1, E2 and E3, with the latter subunit also a constituent of the pyruvate dehydrogenase and ketoacid dehydrogenase complexes. Therefore a defect with the E3 subunit can affect all three enzymes, resulting in a more complicated clinical phenotype.

We show that simulations using our model can replicate the disease state phenotypes of these disorders by correctly predicting the effects of these perturbations on normal mitochondrial metabolism. The simulations show how alternative metabolic pathways can maintain production of ATP, but lead to the over-production of other metabolites that must be excreted, as observed *in vivo *in the blood, urine and cerebrospinal fluid of patients. Furthermore, the simulations suggest that treatments, such as a low glucose diet or amino acid supplements, may reduce the risk of lactic acidosis and increase ATP production and thus be of therapeutic value.

## Results

### Construction of the mitochondrial metabolic model

The metabolic model of reactions in a human heart mitochondrion, called *i*AS253 (designated using a common naming convention for metabolic networks [27]), was constructed using the principle of metabolite availability, where a reaction was only included in the model if the substrates or products of the reaction were used elsewhere in the mitochondrial matrix (See Methods for full details). In total 2187 reactions with evidence of mitochondrial localisation were evaluated resulting in the inclusion of 229 reactions that formed a connected network using 228 metabolites in the mitochondrial matrix compartment. An additional 24 cytosolic reactions were added to a separate compartment to represent reactions that were intrinsic to the function of the mitochondrion, such as glycolysis. 73 boundary conditions were included to represent import and export of metabolites to the system.

To more accurately reflect the *in vivo *capabilities of the mitochondrion and to remove all thermodynamically disallowed flux loops from the model, 110 of the 258 reactions and 50 of the 89 transport steps in the model were assigned a directionality constraint. Reaction directionality was set using thermodynamic information, general rules of irreversibility, and information from several public resources and the primary literature.

The final model contained no orphan metabolites and all internal flux loops were eliminated. 247 reactions were capable of having a flux under the current boundary conditions between the cytosol and the matrix, with the remaining 6 reactions requiring the import of acetaldehyde or propanoate to have a flux, which is currently disallowed in the model. Details of the reactions, metabolites and constraints included in the model can be found in Additional Files 1, 2 and 3. The XML model of *i*AS253 (Additional File 4) has been deposited at the EMBL European Bioinformatics Institute's BioModels database (ID: MODEL1106160000).

### Simulation of normal mitochondrial physiology in cardiomyocytes

Six separate pseudo reactions were devised to denote the metabolite requirements of the mitochondrion. This included the production of 20 amino acids required for protein synthesis, RNA and DNA, haem, ATP and lipids in a ratio found in the inner mitochondrial membrane of rat liver mitochondria (phosphatidylcholine 40%, phosphatidylethanolamine 34%, phosphatidylserine 3%, cardiolipin 18% [28]). Separate simulations were run setting each of these pseudo reactions as the objective function (i.e. maximising the flux through the reaction) and the model was capable of fulfilling all of them. For example, with simulations using maximum ATP production as the objective function, core metabolism worked correctly with both glucose and lactate converted to pyruvate in the cytosol as appropriate for heart [29], which was imported into the mitochondrion to take part in the TCA cycle. NADH was produced by the TCA cycle in preference to fatty acid and ketone body oxidation although these processes were still active, and was used by the reactions representing the electron transport chain to move protons into the cytosolic compartment. Reactive oxygen species (ROS) were produced from complex I at a rate corresponding to 0.1% of the electrons passed through it, as is consistent with recent experimental findings [30,31]. ATP synthase allowed these protons to move back into the mitochondrial matrix compartment, generating ATP in the process. The maximum ATP production in this simulation was 139.43 μmol/min/gDW, which is similar to the experimentally measured figure of ATP hydrolysis of resting heart, which is approximately 150 μmol/min/gDW [29]. Both oxygen and glucose uptake were at the maximum allowable rate (19.8 μmol/min/gDW [32] and 0.9 μmol/min/gDW [33] respectively) and the largest fluxes occurred through the protein complexes of the electron transport chain. When the results of this simulation are compared to experimentally determined figures, the TCA cycle had a flux rate of approximately 7.13 μmol/min/gDW, compared to the experimentally measured figure of 7.5 μmol/min/gDW in rat [32] while the fatty acid oxidation rate of 0.41 μmol/min/gDW is similar to the experimentally determined rate in rat of 0.35 μmol/min/gDW [34]. Very low levels of alanine, succinate, and glutamine were effluxed from the system, corresponding with observations of mammalian heart [35-39]. In addition 61% of acetyl-CoA production was derived from fatty acid degradation, 30% from glycolysis and the remaining 9% from ketone body degradation, which compares well with estimates in the literature that 60-90% of acetyl-CoA production is from fatty acid degradation with the remaining 10-40% from glycolysis [29].

### Simulation of mitochondrial disease: 1) Fumarase deficiency

One of the most noticeable effects of fumarase deficiency is the presence of fumarate in the urine. However, in the default model there was no mechanism for the efflux of fumarate from the system. Therefore, to model fumarase deficiency it was necessary to add an unconstrained transport step and boundary condition to allow fumarate in the mitochondrial matrix to leave the system. Separate simulations were run under each of the six objective functions with this adapted model and no flux was observed through the transport step or boundary condition under any simulation under normal conditions, showing that these additions did not affect the model.

**Figure 1:**
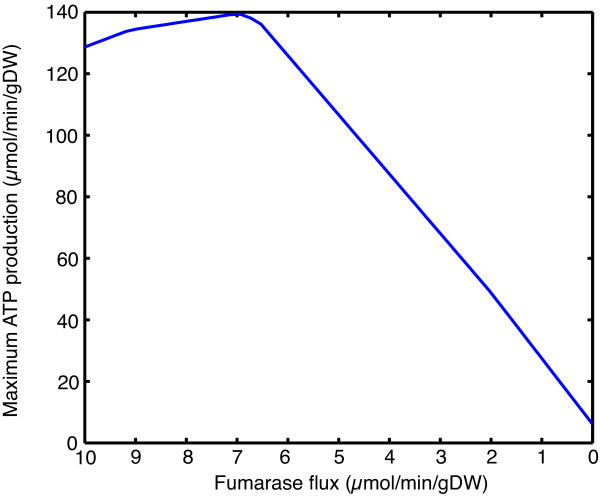
**Effect of varying fumarase flux on maximum ATP production**. The peak in ATP production at a fumarase flux of approximately 7 μmol/min/gDW represents the optimum rate observed under normal conditions.

To model fumarase deficiency the reaction that represents the fumarase enzyme in the model (R01082MM) was constrained to different flux values representing reduced enzyme activity and an objective function of maximum ATP production used. Compared to simulations under normal conditions, the most notable effect of these constraints was a dramatic drop in maximum ATP production of up to 96% (Figure 1). Fumarate was effluxed from the system when the fumarase flux was constrained to just 97% of its value under normal conditions, while lactate was effluxed at 20% of normal flux or below. Flux through the fatty acid oxidation pathway and ketone body degradation became inactive at 28% and 95% of normal fumarase flux respectively, due to inability of the acetyl-CoA produced to enter the TCA cycle, resulting from an inadequate supply of oxaloacetate that would normally be derived from fumarate.

**Figure 2:**
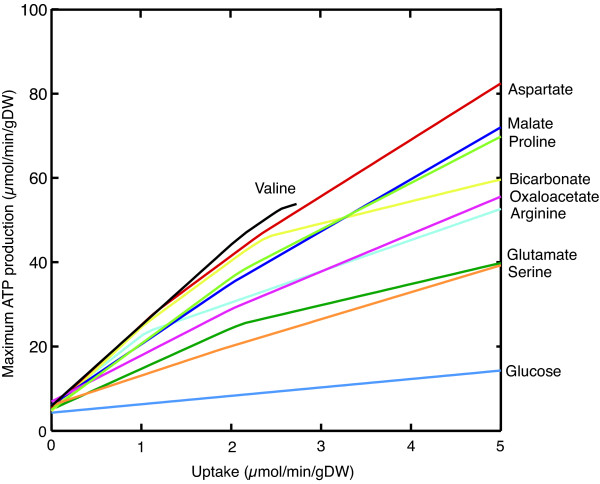
**Effects of metabolite uptakes on maximum ATP production while fumarase reaction flux is at 0% of its flux under normal conditions**.

**Figure 3:**
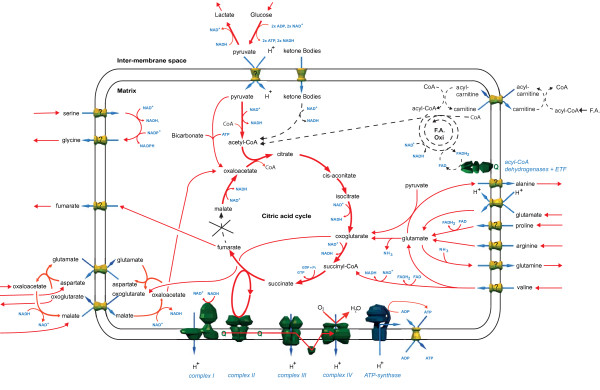
**A summary of multiple simulations showing the entry points into central metabolism of metabolites that increase ATP production during fumarase deficiency when their import is individually unconstrained**. Active pathways are shown in red and inactive pathways in black.

To determine if the import of other metabolites could increase maximum ATP production while fumarase was impaired, the restrictions on each constrained boundary condition were removed in separate simulations. This identified the import of several amino acids, bicarbonate, and metabolites involved in the malate-aspartate shuttle as having a substantial effect on ATP production (Figure 2). In the initial simulations of fumarase deficiency, many of these metabolites were imported at the maximum rate allowed under normal boundary constraints, but these uptake rates had a relatively minor effect on the overall system. Proline, arginine and glutamate were imported and degraded to enter the TCA cycle as oxoglutarate while oxaloacetate, aspartate and malate were imported via the malate-asparate shuttle to circumvent the gap in the TCA cycle caused by the fumarase deficiency. Bicarbonate was used for a metabolic bypass to directly convert pyruvate to oxaloacetate, with the addition of ATP. The routes of these metabolites into central metabolism are shown in Figure 3.

One of the clinical phenotypes of fumarase deficiency is the presence of oxoglutarate in the urine. In the model, an efflux of oxoglutarate from the system was observed when the uptake rates of oxaloacetate, malate, citrate and isocitrate were increased. The efflux was the result of the oxoglutarate, and oxodicarboxylate transporters requiring oxoglutarate to be counter exchanged for the import of the other metabolites into the matrix. However, the uptake of citrate and isocitrate had a small effect on maximum ATP production, increasing it by only 16% when an artificially large uptake rate of 5 μmol/min/gDW was allowed.

### Simulation of mitochondrial disease: 2) Succinate dehydrogenase deficiency

The results from the simulations of succinate dehydrogenase deficiency were very similar to that of fumarase deficiency. The main difference was that when the succinate dehydrogenase reaction (R02164MM) was constrained and an objective function of maximum ATP production used, succinate was effluxed from the system rather than fumarate. A large drop in ATP production was also observed that was slightly more pronounced than fumarase deficiency (Figure S1, Additional File 5), due to the lack of complex II contributing to the quinone pool of the electron transport chain. Fatty acid oxidation and ketone body degradation became inactive and lactate was effluxed at very similar levels of residual enzyme flux to that of fumarase deficiency.

To represent that some patients have some residual enzyme activity, the import of metabolites that had an effect on ATP production was investigated at 33% and 0% of flux of normal conditions. Regardless of the flux level chosen proline, arginine, valine, bicarbonate, aspartate, malate and oxaloacetate were found to have a positive effect on maximum ATP production (Figures S2 and S3, Additional File 5). The entry routes of these metabolites into the TCA cycle were the same as under fumarase deficiency (Figure S4, Additional File 5).

### Simulation of mitochondrial disease: 3) α-ketoglutarate dehydrogenase deficiency

The effect of α-ketoglutarate dehydrogenase deficiency on maximum ATP production was minor. A reduction in ATP production of just 5% was observed when flux through the reaction was constrained to zero and an objective function of maximum ATP production used. This was due to the presence of a bypass that converted oxoglutarate to succinate via succinate semialdehyde and 4-aminobutanoate (GABA) (R01648MM, R00261MM and R00713MM). An efflux of oxoglutarate from the system was observed when the flux through the bypass was constrained by any level. An efflux of lactate was not observed under any of these conditions. To simulate the effect of a defect in the E3 subunit of α-ketoglutarate dehydrogenase that is shared with pyruvate dehydrogenase and ketoacid dehydrogenase enzyme complexes, the dihydrolipoamide dehydrogenase reaction was also constrained (R07618MM). This resulted in the production of lactate, as pyruvate from glycolysis could no longer enter the TCA cycle, but maximum ATP production remained stable, as fatty acid synthesis increased proportionally (up 80%, with an enzyme flux of 0%) to maintain acetyl-CoA production levels.

## Discussion

Here we present a new, manually curated metabolic model of the mitochondrion present in human heart tissue, called *i*AS253 (Additional File 4). It contains 229 mitochondrial matrix reactions, 24 cytosolic reactions, 89 compartment transport steps and 73 boundary conditions. Each reaction was manually evaluated in conjunction with the principle of metabolite availability before incorporation into the model, using 33 mitochondrial proteomic datasets from the MitoMiner database, combined with annotation from public resources and the literature. The model includes directionality constraints based upon general rules of irreversibility, thermodynamics and information from public resources and the literature. The resultant model contains no orphan metabolites and its directionality constraints allow every reaction in model to potentially possess a flux, while all flux loops have been eliminated. It is encoded in SBML format [40], and uses KEGG [41] identifiers where possible. Therefore, this model should be readily adaptable for use by other modellers either in isolation or with its addition to models of cellular metabolism. We believe it is the most refined model of the mitochondrion currently available as demonstrated by simulations of normal conditions, which closely correspond with experimentally determined flux figures and metabolic observations of heart. For example, fluxes though the TCA cycle and fatty acid oxidation pathway while using the objective function of maximum ATP production corresponded with measurements in rat heart (7.05 vs. 7.5 and 0.41 vs. 0.35 μmol/min/gDW respectively). The similarities between the model and the results of experiments *in vivo *suggest that the modelled flux distribution of core metabolism is biologically relevant. The minor discrepancies between the flux figures may be due to imprecise boundary constraints, the biological objective *in vivo *for core metabolism not being described accurately by the model's objective function of maximum ATP production, or difficulties in measuring these values experimentally.

These results are in contrast to those of previous mitochondrial FBA models [2,3] where a much higher uptake of oxygen (about 40 nmol/min/gDW) was permitted, while maximum ATP production was 60% lower when this was set as the objective function. This discrepancy may be due to the use of capacity constraints on the transport steps between the cytosol and mitochondrion in these models. These constraints were derived from uptake rates determined experimentally *in vitro*, on isolated carrier proteins in liposomes, so are unlikely to reflect the uptake rates *in vivo *for the mitochondrion. Our model does not use capacity constraints on these transport steps and instead constrains the system at the boundary conditions.

The constraints placed on the boundary conditions of the system critically affected the behaviour of the model. These constraints reflect the imports and exports of the system and were chosen carefully from the primary literature (Additional File 3). There is some uncertainty in these figures as often the only data available are not from human and in one case not from heart tissue. In addition many of these experiments were conducted on isolated hearts, which may display significant metabolic differences to hearts *in situ *[42]. Therefore these figures may not correspond to the maximum possible uptake values, which are necessary to set the upper bound of the constraint. Previous reconstructions have tried to account for this experimental uncertainty by increasing these figures by an arbitrary amount, such as 25% [2]. This is problematic because no data exists on what scale of increase is appropriate whereas the uptake of some compounds, such as oxygen, are a critical limiting factor to the system under most circumstances, and will usually be at the maximum allowable rate. Therefore in the absence of more relevant data it was decided to use experimental figures without modification. Further experiments to determine and verify the uptake rates used would be beneficial for further refinement of the model and in particular to aid simulations of perturbed states where uptakes can have a large impact on model behaviour. In addition we included directionality constraints on the transport steps between the cytosol and the mitochondrial compartments to prevent flux loops, while reflecting the biological role of the underlying carrier and allowing normal metabolism. However, it is possible that under disease conditions the accumulation of metabolites may affect the direction of transport. In the metabolic disorders investigated here all metabolites that were experimentally reported as accumulating could be effluxed without the need to alter these constraints. The only exception was fumarate, which is known to accumulate in fumarase deficiency, where an extra transport step was included to allow its efflux.

A common objective function used with models of microorganisms is the production of biomass. Biomass represents all the metabolites that are required for growth of that particular organism, with the assumption that the organism is biologically optimised for maximum growth. The relative proportions of each metabolite in the biomass are derived from experimental figures measured from cellular biomass. However, a single biomass objective function cannot currently be devised for the mitochondrion, as no experimental figures exist for its composition. It is also unlikely that the biological objective *in viv*o is maximum growth as one of the main roles of the mitochondrion is to produce ATP and so this would also have to be incorporated in the objective function to make it biologically relevant. This would require the ratios between ATP production and growth and maintenance of the mitochondrion to be experimentally verified and this is not currently available, and is likely to be extremely difficult if not impossible to determine. Therefore to represent the different aspects of mitochondrial metabolism, six separate pseudo reactions were devised, encompassing growth and maintenance as well as haem and ATP production. Although maximum ATP production is most likely to be the closest to the biological objective (as shown with the close correspondence to experimentally measured figures), the inclusion of the other pseudo reactions ensures the model can produce metabolites known to be required by the mitochondrion. However the flux distributions found when using these other pseudo reactions individually as objective functions are unlikely to be realistic, as no one objective function in isolation will match the biological objective of the system *in vivo*. The pseudo reactions that are used for these objective functions have been included in the model for convenience and to allow other researchers to easily replicate our results.

To demonstrate the predictive abilities of the model we simulated and analysed the complicated phenotypes of three disorders of the TCA cycle using the *i*AS253 model.

### The mechanism of fumarase deficiency

Patients with the most severe symptoms of the disorder often have residual fumarase activities near zero [22]. This would equate to near zero flux through the fumarase reaction in the model. Under these conditions both lactate and fumarate were effluxed from the system, matching the phenotype. Initial simulations would predict that such a reduction would result in a very large decrease in maximum ATP production in heart (Figure 1), which would seem likely to have catastrophic effects. However, heart defects in patients have not been reported [22], which would imply that the predicted reduction in ATP production must be compensated. In a series of simulations the boundary constraints were removed one by one to determine if increased metabolite uptake could counteract the decrease. This identified the uptake of valine, aspartate, malate, proline, bicarbonate, oxoaloacetate, arginine, glutamine, serine and glucose as having a positive effect on ATP production (Figure 2).

The effect of aspartate on the system supports the hypothesis of Bourgeron *et al*. [13], which suggests a reaction in the malate-aspartate shuttle is used to convert aspartate and oxoglutarate into oxaloacetate and glutamate, acting as a metabolic bypass and allowing the impaired part of the TCA cycle to be circumvented. They suggest the low level of aspartate found in the blood plasma of two patients in their study supports the existence of such a bypass. The *i*AS253 model predicts that if aspartate is available, this bypass is used. A relatively small uptake resulted in a large difference in maximum ATP production, which suggests it could be biologically relevant.

Oxaloacetate and malate also had a large effect by using the reactions of the malate-aspartate shuttle. In the simulations oxaloacetate was converted to malate before being transported to the matrix using the oxoglutarate transporter. Malate was then converted back into oxaloacetate to complete the TCA cycle. As a consequence of the transport step oxoglutarate was counter-exchanged from the matrix into the cytosol and then effluxed from the system. Such a mechanism *in vivo *may explain the oxoglutarate observed in urine of patients with fumarase deficiency.

The import of proline, arginine, glutamate and valine caused a large increase in ATP production due to the degradation of these metabolites into oxoglutarate, increasing the flux through part of the TCA cycle, and leading to an increase in fumarate efflux. The model predicts that a corresponding increase in the efflux of glutamine and alanine should also be observed. The production of glutamine was required to remove the ammonia produced during the deamination of these metabolites, whereas alanine efflux was involved in the glucose-alanine cycle, where pyruvate is converted into oxoglutarate (Figure 3). However, both of these metabolites are further metabolised in the kidney and liver respectively, so it is not clear whether they would accumulate to a sufficient degree to be elevated in a patient metabolite profile.

The uptake of bicarbonate had a large impact on ATP production by allowing the direct conversion of pyruvate to oxaloacetate. However, due to the production of lactate, and bicarbonate acting as a cellular buffer, it may not be available at sufficient levels to have a significant affect *in vivo*.

Serine had an effect on increasing ATP production, but only from its degradation to glycine, producing NADH and NADPH in the process. It did not directly contribute to the TCA cycle, which explains why even at high uptakes its effect on maximum ATP production is lower than the other metabolites except glucose.

Although increased glucose uptake did increase ATP production the effect was small, while dramatically increasing lactate efflux. Therefore, although increasing glucose levels may be easily achieved physiologically, a low glucose diet, while increasing the levels of the other metabolites may be more beneficial to maintain ATP production, while minimising lactic acidosis.

It is not apparent if *in vivo *the uptakes of any of these metabolites are increased beyond normal limits and have a significant impact on ATP production. Although it may be unlikely that any one metabolite could sustain a high uptake flux under physiological conditions, it may be feasible that all of these metabolites contribute collectively to increasing ATP production. Further experimental data would be required to test these predictions and to further refine the model.

A common occurrence throughout these simulations was fatty acid metabolism was restricted, as acetyl-CoA could not enter the TCA cycle. If this occurs *in vivo*, these excess fatty acids may be stored in adipocytes or alternatively be expressed as an increase in the production of ketone bodies (directionality constraints prevent this in the current model as only liver produces ketones while the heart consumes them), a known cellular method to use excess acetyl-CoA, rather than a reduction in fatty acid metabolism. This would be supported by the elevated levels of acetoacetate in the blood plasma of two patients with the disorder (0.07 and 0.08 mmol/l) in comparison to controls (0.016 - 0.04 mmol/l), although levels of hydroxybutanoate appeared to be within the normal range [13]. Additional data from fumarase deficient patients are needed to verify if these predictions are correct.

### The mechanism of succinate dehydrogenase deficiency

The simulations of succinate dehydrogenase deficiency were very similar to fumarase deficiency in both the reduction in ATP production (Figure 1 and Figure S1, Additional File 5) and the effect of increasing certain metabolite uptake rates (Figure 2 and Figures S2 and S3, Additional File 5). The metabolites that had a positive effect on maximum ATP production were shared between the two deficiencies and used the same degradation pathways and entry points into the TCA cycle (Figure 3 and Figure S4, Additional File 5). The main difference was the efflux of succinate rather than fumarate as found in fumarase deficiency and a slightly lower level of ATP production, as complex II could no longer contribute to the electron transport chain. The similarities between the disorders are unsurprising as the enzymes are adjacent in the TCA cycle. The main physiological difference between them is that many patients with succinate dehydrogenase deficiency have residual enzyme activity in the range of 10-50% whereas many patients with fumarase deficiency have 0% activity [22]. However, it is not possible to predict what level of succinate dehydrogenase flux this will correspond with, as residual enzyme activity and enzyme flux are not directly correlated [43]. However, even low levels of enzyme flux result in a dramatic increase in maximum ATP production (Figure S1, Additional File 5). This has the effect that the uptake rates of metabolites that impact ATP production can be much lower while returning production to normal levels (Figure S2, Additional File 5), which may make these interventions more physiologically feasible.

### The mechanism of α-ketoglutarate dehydrogenase deficiency

The effect of α-ketoglutarate dehydrogenase deficiency on maximum ATP production was minor even when no flux was allowed through the reaction representing the enzyme. This was the result of a bypass known as the GABA shunt [44], which converts oxoglutarate to succinate using succinate semialdehyde and GABA as intermediates, circumventing the impaired reaction. Although the GABA shunt is usually associated with brain, there is evidence in MitoMiner, and the literature [44] that it is present in heart. Oxoglutarate was only effluxed from the system if the flux through the GABA shunt was limited so that not all the flux could proceed through it. If the bypass *in vivo *is unable to sustain the large flux required for the TCA cycle to function at full capacity, this could explain the phenotype of oxoglutarate excretion. Gene expression data for patients with the disorder may give some indication whether the enzymes that constitute the GABA shunt are up-regulated in heart to allow the bypass to function. GABA also acts as an inhibitory neurotransmitter and its involvement in the bypass may begin to explain the encephalopathy reported in patients.

An efflux of lactate was never observed when only α-ketoglutarate dehydrogenase was constrained. Therefore to determine whether a defect in subunit E3 of the enzyme complex, which is also shared with pyruvate dehydrogenase and ketoacid dehydrogenase complexes, was responsible, the reactions representing the latter were also restricted. Only when pyruvate dehydrogenase was constrained was lactate produced, as not all pyruvate from glycolysis could enter the TCA cycle. This suggests that a lactic acidosis phenotype is the result of a defect in the E3 subunit, and isolated α-ketoglutarate dehydrogenase deficiency can be discounted.

## Conclusions

We have successfully simulated metabolism of heart mitochondria under normal conditions as demonstrated by close similarity of flux values with experimentally measured figures. We show that the model also has the ability to simulate the metabolic phenotype of three rare single gene disorders and illustrate that the model can provide an insight into the disease mechanism. As the mitochondrion is involved in many more complicated disorders including Parkinson's, diabetes and heart failure, the next step is to use this model with further development to simulate and understand these conditions. We believe that our model can be used as framework for integrating genomics, proteomics, metabolomics and clinical data and so contribute to the development of therapies for mitochondrial related diseases.

## Methods

### Compartmentalisation and modelling approach

The mitochondrion consists of four distinct components: the outer mitochondrial membrane, the intermembrane space, the inner mitochondrial membrane, and the mitochondrial matrix. For modelling metabolites, no distinction is required between the intermembrane space, outer mitochondrial membrane and the cytosol, as porins in the outer mitochondrial membrane allow any molecule below 3-6 kDa to exchange freely between the cytosol and the intermembrane space. In contrast the inner mitochondrial membrane is impermeable to most metabolites [45] and the exchange of metabolites between the mitochondrial matrix and the cytosol requires a transporter in the inner mitochondrial membrane [46]. Therefore, the model has two compartments, one to represent the cytosolic side of the intermembrane space and another for the mitochondrial matrix, with the model focused on metabolic reactions in the latter compartment. Reaction localisation was determined using the principle of metabolite availability, where a reaction can only be present if its substrates are available and its products can be used within the same compartment.

### Metabolite availability from mitochondrial transporters

Initial metabolite availability within the mitochondrial matrix was determined with the addition of inner mitochondrial membrane transporters to the model and defining the metabolite requirements of the mitochondrion. The inner mitochondrial membrane transporters represent the members of the mitochondrial transporter family, many of which are characterised and their substrates and transport mechanism (i.e. uniport, antiport, symport, proton coupled) are known [46,47]. Some carriers can transport many related metabolites, so each possible combination was separately included into the model with the appropriate transport mechanism. Each transport step was modelled as a reaction where only the subcellular localisation of the metabolites changed.

### Addition of known mitochondrial matrix reactions

The next step in defining metabolite availability was the addition of reactions known to take place in the mitochondrial matrix (Additional File 1). From the MitoMiner database all proteins with an Enzyme Commission (EC) number that had annotation in either UniProt [48] or the Gene Ontology [49] as localising to the mitochondrial matrix were identified. MitoMiner was used to find the corresponding reactions in KEGG for each EC number. Every reaction was manually inspected to uncover any erroneous annotation by cross-referencing with the experimental data from MitoMiner, BRENDA [50] and the primary literature to confirm the protein was present in human, expressed in heart tissue and localised to the mitochondrial matrix. If the reaction met these criteria it was added to the model. This process was repeated for proteins annotated as localising to the inner mitochondrial membrane. The identified reactions were assigned to a compartment based on which side of the inner mitochondrial membrane the protein has its active site. If this information was unavailable it was evaluated under the criteria used in model extension section, below. To ensure that all known mitochondrial matrix reactions were included, the reactions of established mitochondrial processes, such as amino acid degradation, were obtained from KEGG and included if absent.

### Model extension via metabolite availability

The model was extended by identifying orphan metabolites that were used in only one reaction and searching for further reactions that could produce or consume them. Each new reaction was evaluated for localisation using information from MitoMiner, UniProt, the Gene Ontology, BRENDA and the primary literature. If the evidence and annotation for mitochondrial localisation was convincing (Additional File 1), or if the reaction was spontaneous, it was added to the model. If this addition created a new orphan metabolite, the process was repeated with the new metabolite. As reactions that use orphan metabolites are unable to have a flux and so do not contribute to the model, if a suitable reaction could not be found to produce or consume an orphan metabolite in the mitochondrial matrix, the metabolite was considered to be unavailable and the reaction that generated it was removed from the model. In exceptional cases where an orphan metabolite had strong evidence that it was present within the mitochondrial matrix but used or produced outside it, such as pyruvate, or was required to fulfil an objective function, such as an amino acid, then a transport step was added to the model. Once all the orphan metabolites had been resolved, all remaining reactions that had evidence or annotation in MitoMiner for mitochondrial localisation were manually evaluated using the same procedure and added to the model where appropriate.

### Cytosolic reactions and boundary conditions

Although the model was focused on the mitochondrial matrix, some cytosolic reactions were incorporated. These included reactions representing the cytosolic side of the malate-aspartate, glycerol-phosphate and carnitine shuttles as well as the cytosolic steps required for haem synthesis. As pyruvate uptake rates into the mitochondrion are unavailable, but the cellular uptake of glucose has been measured experimentally, the reactions of glycolysis were also included. Finally, boundary conditions constrained to experimentally derived figures from the primary literature (Additional File 3) were added to the model to reflect the import and export of metabolites into the system from the cytosol of both common metabolites and the metabolite orphans present in the cytosol compartment. In some cases the only information available was from non-human species or non-heart tissue types.

### Reaction protonation

All the reactions included were protonated at the pH of either the matrix or the cytosol. The pH of the mitochondrial matrix differs markedly from that of the cytosol resulting in a difference of protonation state for some metabolites, particularly those containing phosphate groups. The protonation state is particularly relevant for a mitochondrial model as changes in free protons can affect the electron transport chain and, therefore, the maximum ATP objective function. As all reactions in KEGG are at neutral pH, the reactions in the model were protonated according to the major microspecies found at a pH of 8.05 for the mitochondrial matrix [51] and 7.3 for the cytosol. These calculations were performed using the Marvin suite of computational chemistry software (ChemAxon Ltd, Budapest, Hungary). Any ambiguous 'R' chemical groups present in the compounds were ignored for the purposes of these calculations. The stoichiometry of the protonated reactions used in the model can be found in the supplementary information. In addition, the stoichiometry of ATP synthase was altered to reflect the latest structural and biochemical information [9].

### Objective functions

Six pseudo reactions were defined to represent the metabolite requirements of essential mitochondrial processes: the availability of 20 amino acids for protein synthesis, nucleotides for DNA and RNA synthesis, lipids in the ratio present in the inner mitochondrial membrane for lipid synthesis and the production of ATP and haem. These pseudo reactions were included in the model and used individually as the objective functions in simulations. For example maximising the production of ATP.

### Model format and in silico analysis

The model was encoded in SBML format and validated using the SBML validator tool [52]. KEGG identifiers were used where possible for the identifiers of reactions and metabolites so they could be easily identified and to fulfil MIRIAM [53] requirements. To denote the compartment of each reaction and metabolite the following were appended to the KEGG identifier: 'MM' for mitochondrial matrix, 'Cyto' for cytosolic and '_b' for boundary. The metabolic capabilities of the model were calculated using flux balance analysis. The six defined objective functions were used in flux balance analysis to find unique flux distributions of the model under different conditions. All simulations were carried out in MATLAB (MathWorks, Inc., Natick, MA) using the COBRA toolbox [54] and the linear programming solver GLPK (http://www.gnu.org/software/glpk). Networks were visualised using Cytoscape [55]. All graphs were generated using the COBRA toolbox. All reported flux values are μmol/min/gram of dry weight (DW).

### Reaction reversibility and directionality constraints

Reversibility of all the reactions was manually evaluated and a direction assigned using general rules of irreversibility [56] and estimates of ΔG determined using the group contribution method [57]. If none of these criteria were applicable the reaction was considered to be reversible. Details of each assignment can be found in Additional File 1.

Additional constraints on the directionality of the reactions and transporters in the model were applied to prevent thermodynamically disallowed flux loops occurring, known as type II and III extreme pathways [58]. Internal flux loops were identified by running a simulation under each objective function while all boundary conditions were constrained to zero, preventing any influx or efflux of metabolites to the system. Under these conditions all fluxes in the system should be zero with any observed fluxes the result of loops. Loops were eliminated with the addition of directionality information from the primary literature, KEGG, BRENDA and HumanCyc [59]. Where necessary, transport steps in the model were also constrained to one direction to prevent loops. These were particularly prevalent as many of the transport steps use a similar set of metabolites as they represent different metabolite combinations that are capable of being transported by the same mitochondrial carrier. As thermodynamics and rules of directionality cannot be applied because the free energy change of exchange is zero under standard conditions, they were constrained to reflect the biological role of the carrier. For example as the function of the oxoglutarate carrier is to exchange cytosolic malate for oxoglutarate in the matrix as part of the malate-asparate shuttle [46], this directionality can be applied to the transport step. Additional simulations were conducted to ensure each reaction in the model was still capable of having a flux with these directionality constraints, with alterations where necessary, while still eliminating loops.

To prevent unrealistic flux distributions from the unlimited uptake or efflux of metabolites, the fluxes through the boundary conditions were constrained. To determine which boundary conditions had a critical effect on the model and so required constraining, all boundary conditions were set to an arbitrarily small upper bound value of 0.01 μmol/min/gDW. Each boundary condition was unconstrained one by one and simulations were run for each objective function. Any large influx or efflux from an unconstrained boundary condition was investigated and constrained to experimentally determined values obtained from the primary literature with conversions to the correct units (μmol/min/gDW) where appropriate. A wet to dry weight conversion of five times was used where required, based upon the study of isolated mouse hearts [60]. In some cases experimental figures were unavailable but directionality could be assigned to the boundary condition to prevent an unrealistic flux. Finally a simulation under each objective function was carried out to determine whether the fluxes representing core metabolism reflected that found *in vivo*. If core metabolism was disrupted by the import of a metabolite, the unconstrained boundary condition was identified and constrained to an experimentally determined value or directionality.

### Modelling of disease

Fumarase deficiency was modelled by constraining the fumarase reaction (R01082MM) to various percentages of its flux under normal conditions. Due to the non-linearity between enzyme activity and reaction flux in networks [43], it is not possible to associate these percentages with residual fumarase activities from patients with the disease [13,14,52,61]. One of the major phenotypes of the disorder is the presence of fumarate in the urine. However, there is no mechanism in the default model for its efflux. Therefore a transport step and boundary condition were added to the model to allow the efflux of fumarate produced in the matrix. No other alterations were made to the default model. To determine the effect of these constraints on fumarase, a simulation was run using each of the six objective functions described previously. Separate simulations were run to determine the effect of un-constraining each boundary condition on the system.

To model succinate dehydrogenase deficiency the reaction that represents the enzyme (R02164MM) was constrained to a maximum of 33% and then 0% of its flux when the objective function was set to maximum ATP production. The 33% figure was chosen to represent that there is some residual enzyme activity in many patients, but as this does not directly correlate with flux, an arbitrary reduction in flux was chosen. No other alterations to the model were made. Simulations were carried out using each of the six objective functions. The effect of un-constraining each boundary condition on the system was then determined in separate simulations.

The disorder of α-ketoglutarate dehydrogenase deficiency was modelled by constraining the reaction flux of the initial oxidation reaction of 2-oxoglutarate (R01700MM) to 33% of its level under normal conditions to represent reduced enzyme activity. Separate simulations was performed where the reaction shared with pyruvate dehydrogenase and ketoacid dehydrogenase complexes (R07618MM) were also constrained to various levels of the normal reaction flux to simulate the effects of a defect in the E3 subunit, which are shared between the complexes. In both cases simulations were carried out using each of the six objective functions.

## Competing interests

The authors declare that they have no competing interests.

## Authors' contributions

ACS conducted the reconstruction, and analyses. AJR conceived the study and participated in its design. Both authors contributed to, read and approved the final manuscript.

## Supplementary Material

Additional file 1 **Reaction details**. Excel file detailing the reactions included in the model including the applied directionality, evidence for mitochondrial localisation and corresponding KEGG identifier, where applicable. Click here for file

Additional file 2 **Metabolite details**. Excel file detailing the metabolites included in the model, the assigned compartment and corresponding KEGG identifier. Click here for file

Additional file 3 **Applied constraints**. Excel file detailing the constraints placed on the boundary conditions and the mitochondrial transport steps. Click here for file

Additional file 4 ***i*AS253 metabolic model in SBML format**. XML file of the *i*AS253 model, in SBML format. Boundary constraints and biomass pseudo reactions are included. Click here for file

Additional file 5 **Supplementary Figures. Supplementary Figure S1**. Graph of the effect of varying succinate dehydrogenase flux on maximum ATP production. **Supplementary Figure S2**. Effects of metabolites on maximum ATP production while succinate dehydrogenase deficiency flux is at 33% of its flux under normal conditions. **Supplementary Figure S3**. Effects of metabolites on maximum ATP production while succinate dehydrogenase deficiency flux is at 0% of its flux under normal conditions. **Supplementary Figure S4**. The active pathways (red) that contribute to ATP production during succinate dehydrogenase deficiency. Black dotted lines show pathways that are inactive. Click here for file
