# Supplementary material for: A metabolic model of the mitochondrion and its use in modelling diseases of the tricarboxylic acid cycle
Source: BMC Syst Biol. 2011 Jun 29;5:102. doi: 10.1186/1752-0509-5-102 (PMC3152903; doi:10.1186/1752-0509-5-102)
Supplement: Additional file 5 — Supplementary Figures. Supplementary Figure S1. Graph of the effect of varying succinate dehydrogenase flux on maximum ATP production. Supplementary Figure S2. Effects of metabolites on maximum ATP production while succinate dehydrogenase deficiency flux is at 33% of its flux under normal conditions. Supplementary Figure S3. Effects of metabolites on maximum ATP production while succinate dehydrogenase deficiency flux is at 0% of its flux under normal conditions. Supplementary Figure S4. The active pathways (red) that contribute to ATP production during succinate dehydrogenase deficiency. Black dotted lines show pathways that are inactive. [file 1752-0509-5-102-S5.PDF]

Supplementary Figures

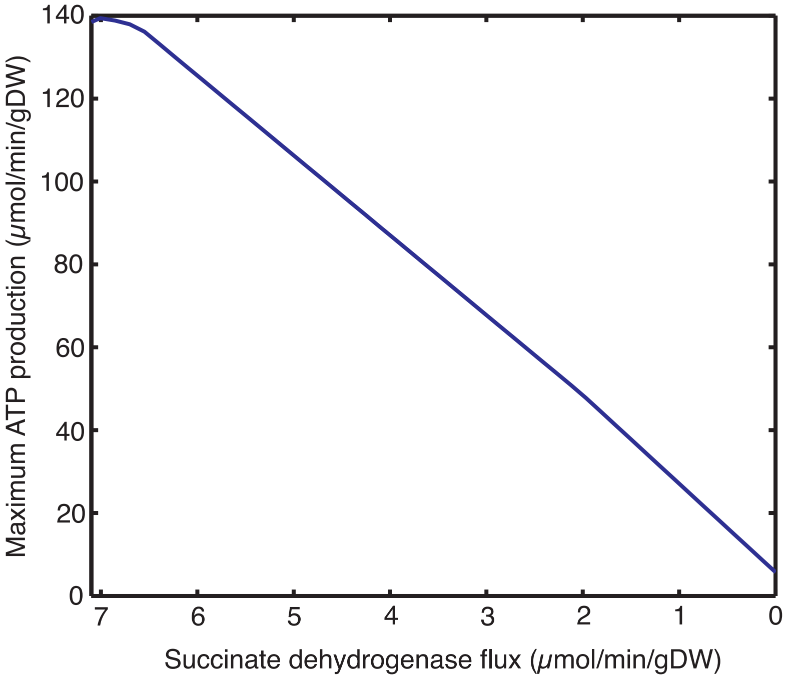

Supplementary Figure S1. Effect of varying succinate dehydrogenase flux on maximum ATP production.

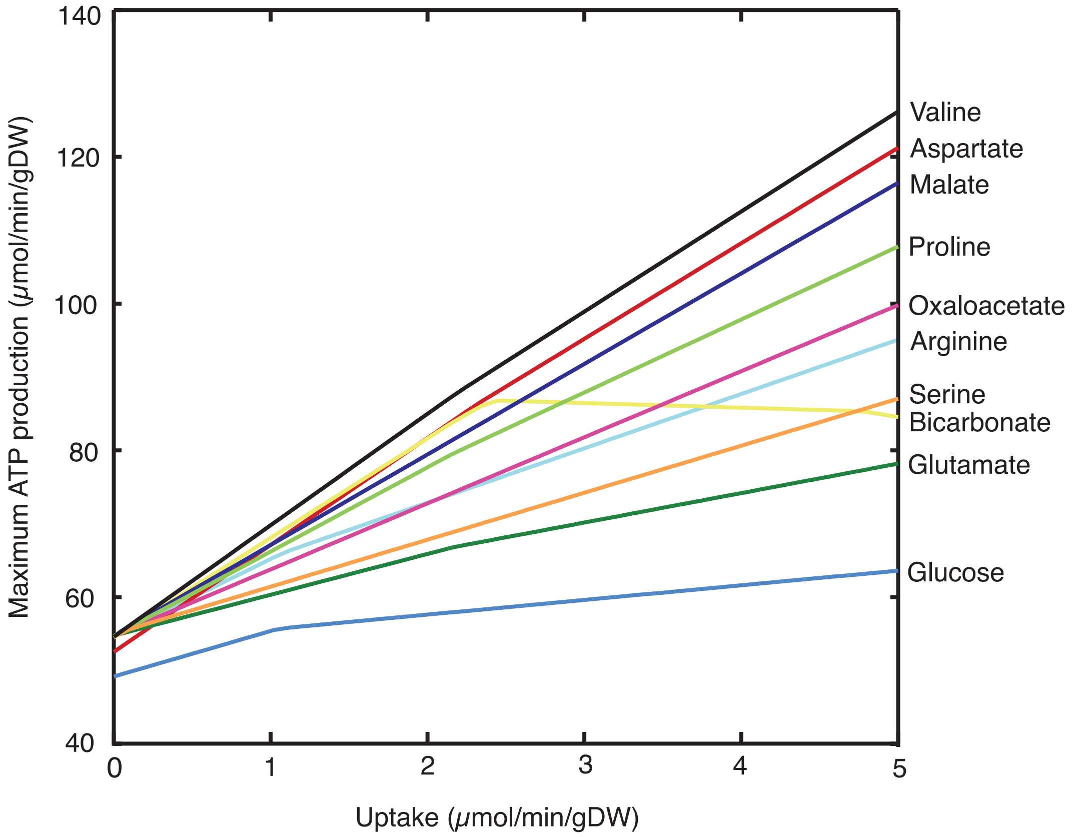

Supplementary Figure S2. Effects of metabolites on maximum ATP production while succinate dehydrogenase deficiency flux is at 33% of its flux under normal conditions.

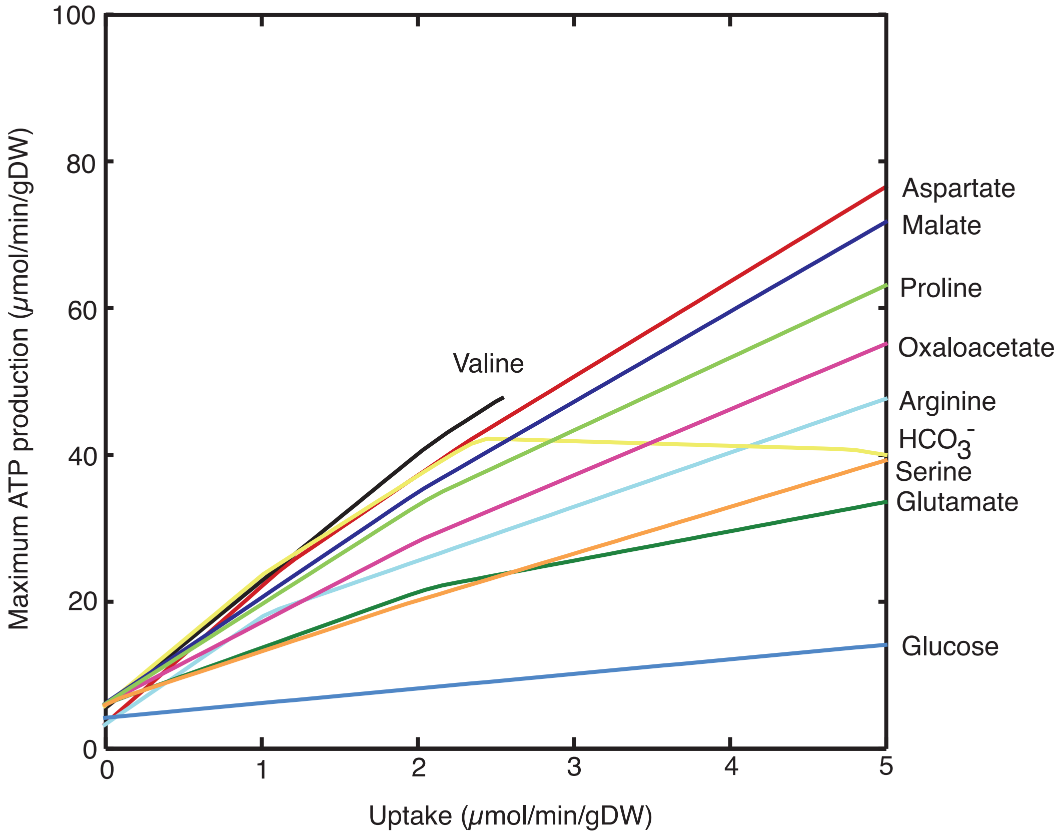

Supplementary Figure S3. Effects of metabolites on maximum ATP production while succinate dehydrogenase deficiency flux is at 0% of its flux under normal conditions.

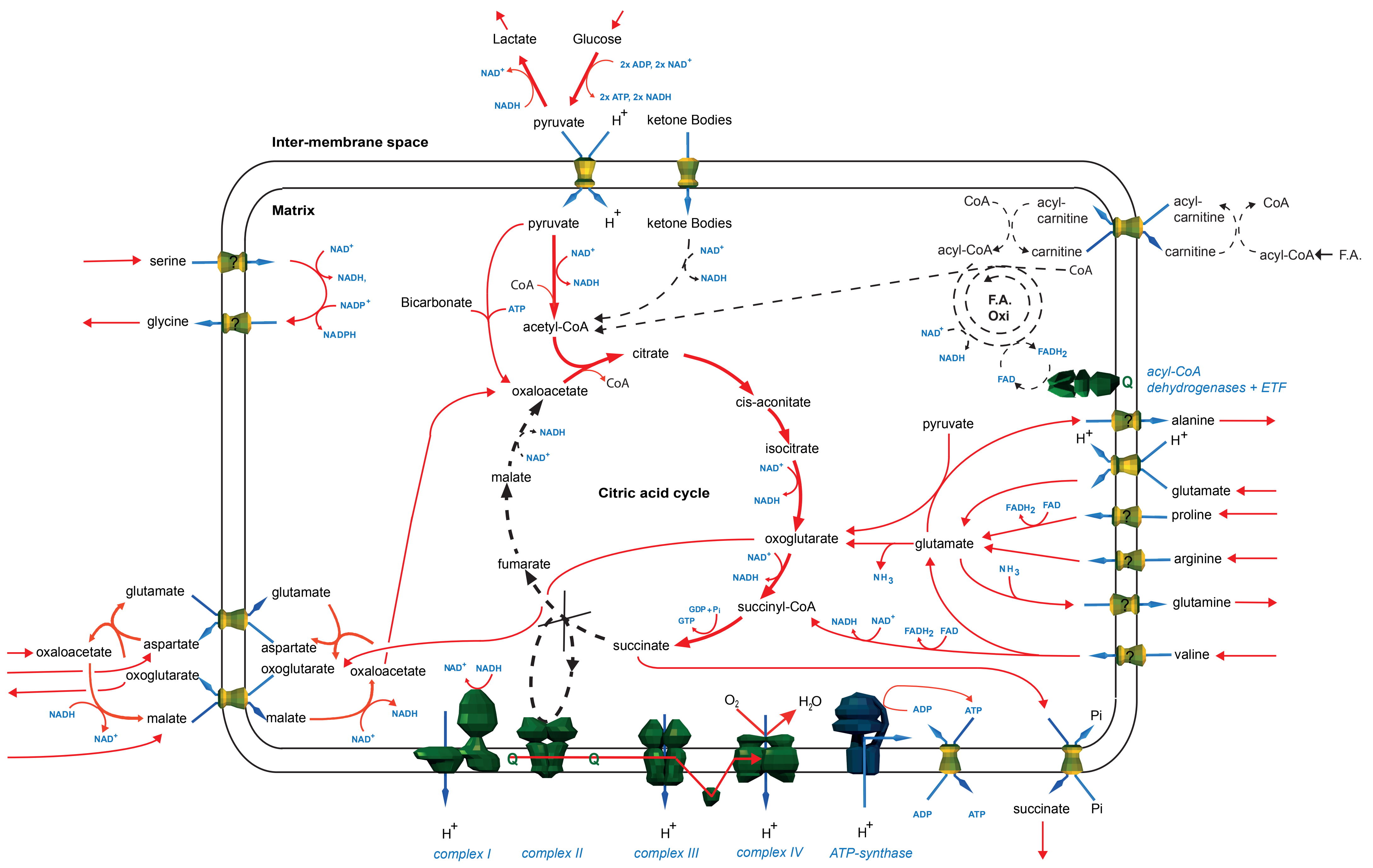

Supplementary Figure S4. The active pathways (red) that contribute to ATP production during succinate dehydrogenase deficiency. Black dot lines show pathways that are inactive.
